# Atherosclerosis in Congenital Heart Disease: Let’s Not Miss the Forest for the Trees∗

**DOI:** 10.1016/j.jacadv.2022.100035

**Published:** 2022-05-25

**Authors:** George Giannakoulas, Andreas S. Papazoglou

**Affiliations:** a First Department of Cardiology, General University Hospital of Thessaloniki AHEPA, Aristotle University of Thessaloniki, Thessaloniki, Greece; b Cardiology Department, Athens Naval Hospital, Athens, Greece

**Keywords:** adults with congenital heart disease, atherosclerotic cardiovascular disease, cardiovascular disease prevention, cardiovascular risk factors, coronary artery disease

**Figure:**
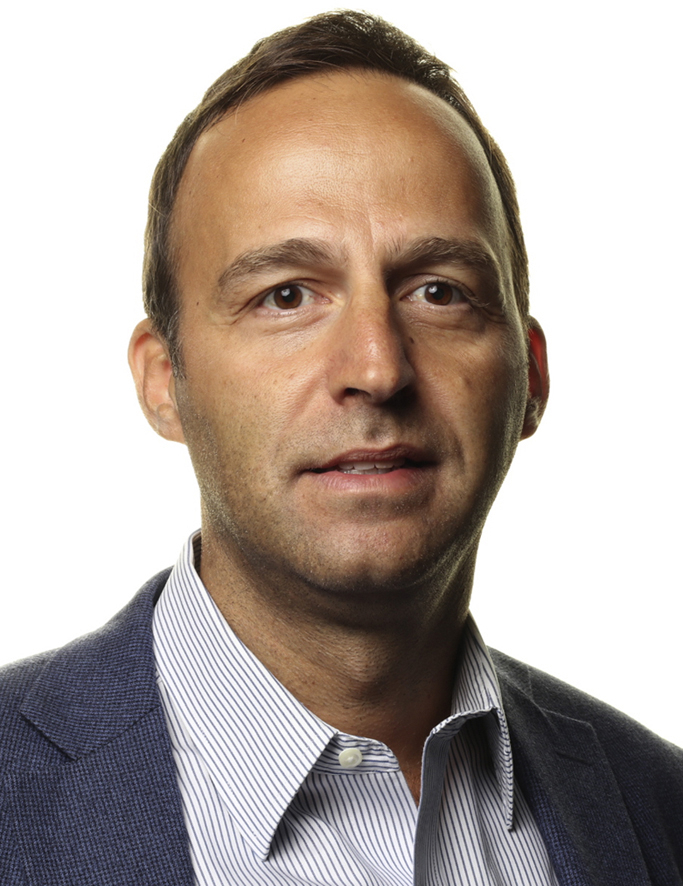

Recent medical diagnostic and therapeutic advances have radically modified the epidemiology of congenital heart disease (CHD), with the vast majority of contemporary CHD patients having a near-normal life expectancy.^1^ However, there has been a shift from mortality from surgery to morbidity, as patients’ longevity translates to a population of adults with CHD faced with acquired conditions, most importantly atherosclerotic cardiovascular disease (ASCVD).^2^ Coronary artery disease (CAD) seems to be a key determinant of outcomes in the elderly adult congenital heart disease (ACHD) population (older than 60 years), while myocardial infarction has become the leading cardiovascular cause of death in the noncyanotic ACHD population.^2^ Despite this threat, awareness of ASCVD and management of lifestyle-dependent ASCVD risk factors continue to be suboptimally addressed in the setting of CHD. In addition, important questions remain unanswered including: 1) whether adverse cardiovascular events in aging ACHD patients are driven by ASCVD or by CHD-related complications; and 2) to what extent do ASCVD risk factors influence the long-term outcome of ACHD.

In this issue of *JACC: Advances*, Egbe et al^3^ provide real-world data to answer these questions and shed additional light on the epidemiology and prognostic significance of ASCVD risk factors in the ACHD population. The authors describe a large cohort of 5,025 ACHD patients (median age: 34 years) without prior CAD and the prevalence and progression of conventional ASCVD risk factors (hypertension, hyperlipidemia, diabetes, obesity, smoking, and family history of premature CAD) within a 5-year follow-up. They also examine the association of ASCVD risk factors with the occurrence of adverse cardiovascular events during a median 6.7-year of follow-up. Regarding the ASCVD epidemiology, 47% of the ACHD patients overall and 77% of elderly (>60 years) ACHD patients had ≥1 ASCVD risk factors at baseline. Almost 15% of the total population developed ≥1 new-onset ASCVD risk factor within 5 years, the most common being obesity and hyperlipidemia. Although 1 out of 5 patients had hyperlipidemia, only 40% of affected patients received lipid-lowering therapy. Both baseline and new-onset ASCVD risk factors were independently associated with the occurrence of adverse events. This relationship was consistent across different CHD diagnoses, anatomical groups, and physiologic stages, despite being less robust in patients with Fontan palliation and those with physiological stage D. Hence, the findings of this large single-center study confirm the hypothesis that the ASCVD risk profile independently affects the clinical course of patients with CHD. Overall, the methodology used to answer these questions was robust with survival analyses adjusted for demographic characteristics, indices of CHD severity, comorbidities, medications, and echocardiographic parameters of ventricular and valvular dysfunction. However, the study had some limitations. Firstly, the cohort studied mostly consisted of subjects with anatomically moderate or complex CHD (93.7% of the sample), possibly due to a specific referral pattern at the Mayo Clinic, and therefore, the generalizability of the study may be restricted even if we do not expect substantial differences in the ASCVD epidemiology for those with more simple CHD anatomy. Secondly, given the retrospective nature of the study, there is potential for unmeasured confounding. ASCVD risk factors may not always be responsible for all cardiac complications, and other variables, such as valvular heart disease, may be the cause of complications such as heart failure. Finally, the investigators did not examine the continuum of ASCVD risk factor exposure using continuous rather than binary variables.

Nevertheless, the study by Egbe et al presents interesting and hypothesis-generating findings which advance our understanding of the importance of ASCVD in the ACHD population. The study demonstrates that the prognostic importance of ASCVD risk factors are consistent across different CHD diagnoses, anatomical groups, and physiologic stages since ACHD populations are remarkably heterogeneous encompassing a multiplicity of diagnoses and stages of complexity. Based on these real-world data, the authors highlight the need for intensified primary prevention of ASCVD in patients with ACHD. We applaud this message and encourage further efforts by the ACHD community to focus on ASCVD prevention.

Findings from the study support existing literature, showing that ASCVD and its risk factors are prevalent, though under-recognized and possibly undertreated in ACHD populations.^4^ It has been shown that 50 to 80% of ACHD patients have ≥1 ASCVD risk factors, depending on their age and CHD complexity.^5^^,^^6^ Hypertension and metabolic syndrome have been reported to occur more frequently in ACHD patients than in age- and gender-matched controls. A sedentary lifestyle along with (unwarranted) self- or physician-restricted exercise, due to perceived risks of underlying CHD or limited capacity for exercise, are key factors contributing to obesity and insulin resistance. Fortunately, the proportion of smoking ACHD patients seems to be smaller than the corresponding general population, possibly because of their health education since birth. Lipids have not been well studied in ACHD populations, and findings are inconsistent.

In the ACHD population, morbidity secondary to acquired ASCVD is becoming more common in parallel with the aging cohort and risk factor development. Traditional ASCVD risk factors have been associated with CAD in several ACHD populations, and ACHD seem to be at a 1.5-fold increased risk for CAD compared to healthy individuals, as indicated by a meta-analysis of 684,200 participants.^7^ The risk of premature CAD may also be increased in ACHD patients due to genetic coronary lesions, postoperative changes, and CHD-related complications beyond acquired atherosclerosis (Figure 1). On the other hand, cyanosis is believed to exert a protective effect on the development of ASCVD. However, most patients with cyanotic CHD have corrective surgery early in childhood (eg, tetralogy of Fallot, transposition of the great arteries) and are no longer cyanotic and therefore lose this protection. One study found that cyanotic CHD patients had a negative lipidemic profile compared to healthy controls, while the subclinical ASCVD burden did not differ between the 2 groups.^8^

**Figure 1:**
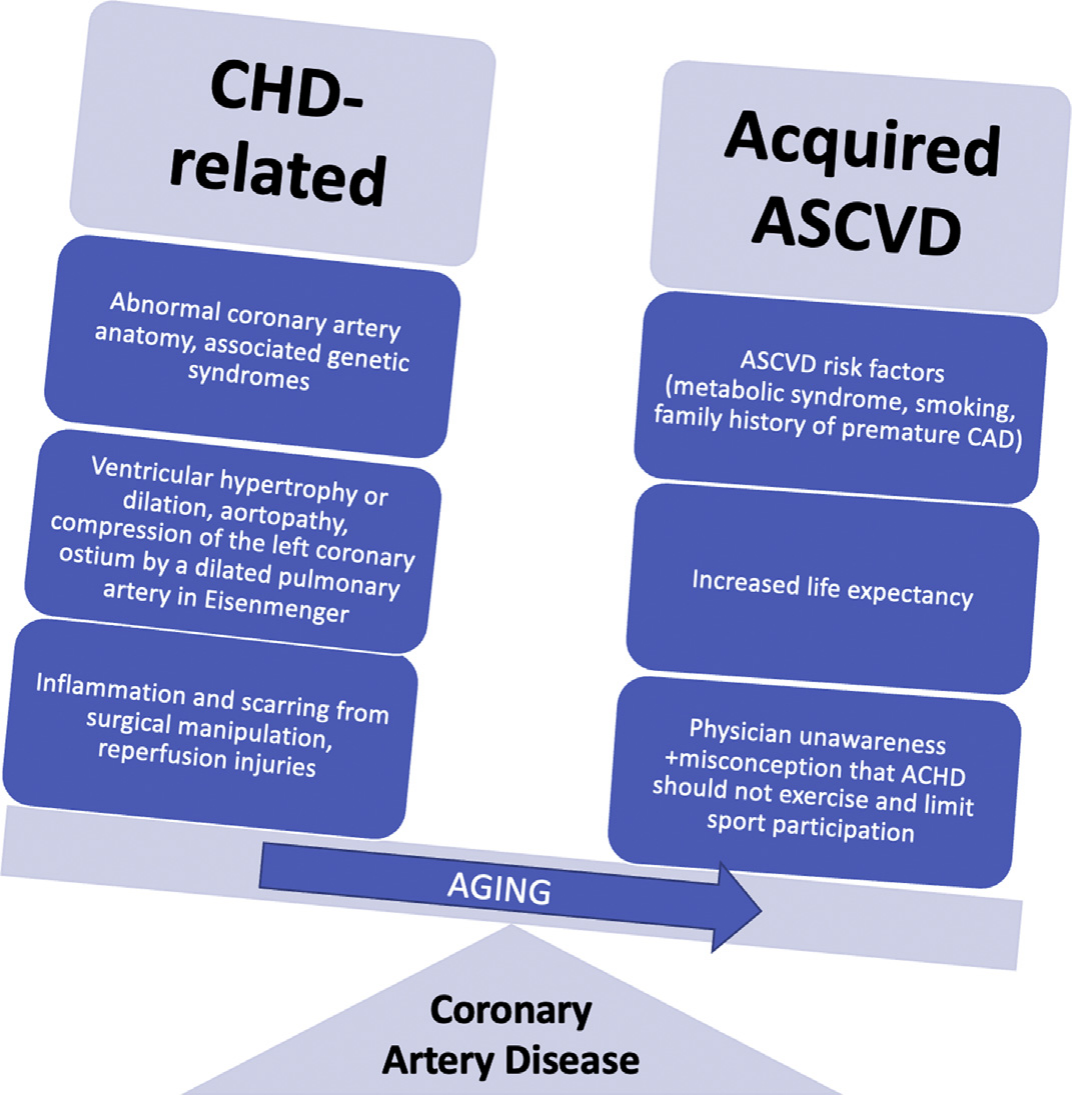
**Potential Mechanisms of Coronary Artery Disease in Adults With Congenital Heart Disease** ASCVD = atherosclerotic cardiovascular disease; CAD = coronary artery disease; CHD = congenital heart disease.

The presence of CHD should not alter efforts to decrease ASCVD risks, irrespective of age and CHD complexity, as is routinely performed in the non-CHD population.^6^ ACHD patients should undergo guideline-based screening and management of modifiable risk factors often best done with a multidisciplinary team approach, including general, congenital, and interventional cardiologists. A healthy lifestyle should be pursued in all ACHD patients, while counseling to encourage daily participation in physical activity and aerobic exercise or cardiac rehabilitation programs (according to functional capacity) should become an essential part of follow-up visits at congenital cardiologist’s offices.^9^^,^^10^ Smoking cessation and adherence to a dietary pattern including reduced calories in saturated and transfatty acids and lower sodium intake should be discussed. Screening for and modifying ASCVD risk factors earlier in life might further improve patient outcomes; hence, pediatric cardiologists also have a crucial role to play in preventive cardiology counseling in children/adolescents with CHD.

There needs to be a paradigm shift toward the holistic management of the ACHD patient, and this requires providers to be cognizant of issues related to both congenital and acquired heart disease. There is also a need for novel ASCVD risk-stratification algorithms in this new era of precision medicine (including imaging parameters, laboratory measurements [eg, hsCRP] and ‘pan-omics’ data) with the potential to identify ACHD that would benefit more from lifestyle or pharmacologic interventions, regardless of baseline ASCVD risk factors. Future research will hopefully lead to new insights into ACHD-specific preventive interventions.

## Funding support and author disclosures

The authors have reported that they have no relationships relevant to the contents of this paper to disclose.
